# Automated Motion Tracking of Vaginal Pessaries and Pelvic Floor Structures on Dynamic MRI

**DOI:** 10.1007/s00192-025-06374-w

**Published:** 2025-10-22

**Authors:** Christopher X. Hong, Mariana Masteling, Kourosh Kalayeh, Jennifer LaCross, John O. L. DeLancey, Luyun Chen

**Affiliations:** 1 Department of Obstetrics and Gynecology, University of Michigan, University Hospital South 1500 E. Medical Center Dr., Ann Arbor, MI L401148109 USA; 2 Department of Biomedical Mechanical Engineering, University of Michigan, Ann Arbor, MI USA; 3 Department of Radiology, University of Michigan, Ann Arbor, MI USA; 4 Department of Urology, University of Michigan, Ann Arbor, MI USA

**Keywords:** Magnetic resonance imaging, Mechanism of action, Motion tracking, Pelvic organ prolapse, Pessary

## Abstract

**Introduction and Hypothesis:**

Vaginal pessaries are a cost-effective, nonsurgical treatment for pelvic organ prolapse (POP), but limited understanding of pessary biomechanics and the inability of static MRI analyses to capture continuous device–tissue interactions hinder design innovation. While dynamic magnetic resonance imaging (MRI) offers insights into pelvic floor biomechanics, conventional analyses rely on static frame comparisons and cannot capture continuous device–tissue interactions. This study aimed to apply a validated, automated motion-tracking framework to dynamic MRI for frame-by-frame analysis of pessary kinematics and evaluate correlations between pessary displacement and changes in hiatus dimensions.

**Methods:**

In this prospective pilot study, six individuals with anterior vaginal wall-predominant POP successfully using a ring pessary with support underwent dynamic 3D pelvic MRI at rest and during maximal Valsalva. A previously validated optical flow-based tracking algorithm was employed to quantify frame-by-frame motion of defined mid-sagittal regions of interest (ROIs), including pessary rims, perineal body, and anorectal angle. These regions of interest were chosen for their biomechanical relevance in capturing the interaction between the pessary and the structures that define the urogenital and levator hiatus size, key determinants of pessary retention. Pearson correlation was used to evaluate the relationship between distal pessary displacement and changes in urogenital and levator hiatus dimensions.

**Results:**

The median age was 66.5 years (range 52–76) and median pessary use was 3 years (range 2–4); all patients used a size 3 or 4 ring pessary and performed self-maintenance. Two of six reported occasional prolapse of the pessary, and all achieved successful retention. Resting MRI showed the pessary positioned posterior and inferior to the pubic bone with elevation of both anterior and posterior vaginal walls in all patients, as compared to resting MRI without the pessary in situ. Automated tracking was successful for all participants. Frame-by-frame analysis demonstrated strong correlations between distal pessary translation and enlargement of the urogenital hiatus (*r* = 0.96 [95% CI 0.94—0.97]) and levator hiatus (*r* = 0.86 [0.81—0.91]). Correlations were stronger than those observed using start-to-end frame comparisons.

**Conclusions:**

This pilot study demonstrates the feasibility of automated frame-by-frame motion to quantify pessary–pelvic floor interactions during physiologic loading. This proof-of-concept establishes a foundation for larger studies to explore patient- and device-specific determinants of pessary function and failure, with the ultimate goal of informing personalized device design and improving clinical outcomes.

**Supplementary Information:**

The online version contains supplementary material available at 10.1007/s00192-025-06374-w.

## Introduction

Pelvic floor disorders—including pelvic organ prolapse (POP) and stress urinary incontinence (SUI)—affect one in four women and can significantly impair physical function, social participation, and quality of life [1]. Vaginal pessaries—prosthetic devices worn in the vagina—are the most cost-effective, nonsurgical treatment for POP [2, 3]. When properly fitted, pessaries restore anatomic support and relieve symptoms of prolapse. However, outcomes remain inconsistent: between 20 and 30% of women with POP cannot be successfully fitted [4, 5]. Among those initially fitted with a pessary, approximately 40% of patients will discontinue use for reasons including pessary pain or discomfort, failure to remain in the vagina, and failure to correct symptomatic vaginal bulging [5].

Although several clinical predictors of pessary failure—such as an enlarged genital hiatus or shortened total vaginal length—have been identified, the fundamental mechanisms underlying pessary function and retention remain poorly understood, limiting meaningful innovation. For instance, a ring pessary is traditionally thought to “support” pelvic organs by positioning behind the pubic symphysis [6]. Recent imaging studies using magnetic resonance imaging (MRI) have challenged this hypothesis, suggesting that the pubic symphysis may play a limited role in pessary mechanics or retention [7, 8]. Furthermore, dynamic MRI has offered new insights into the possible interaction between pessaries and pelvic floor structures [8, 9]. However, a major limitation of current dynamic MRI analyses—which rely on comparing static frames at rest and during maximal Valsalva—is their inability to capture dynamic interactions. Contemporary approaches miss important kinematic information during pessary movement that may help to demonstrate interactions between the pessary and surrounding pelvic floor structures. Currently, no system exists to track the frame-by-frame interaction of pessaries with surrounding pelvic structures, an essential step in elucidating their mechanism of action. Such analysis is not feasible with existing methods, which would require manual identification of relevant structures in every frame of a dynamic MRI sequence.

To address this gap, the objectives of this study were to: (1) apply an automated motion-tracking framework to dynamic MRI to evaluate pessary kinematics during a Valsalva maneuver, and (2) assess the correlation between pessary movement and dynamic changes in urogenital and levator hiatus dimensions. We hypothesized that this motion-tracking framework would enable detailed evaluation of pessary kinematics and that frame-by-frame analysis using this motion-tracking approach would demonstrate a stronger correlation between pessary translation and hiatus size changes than conventional start-to-end frame analysis.

## Methods

We conducted a prospective pilot study involving six patients with anterior vaginal wall-predominant prolapse below the hymen who were using a ring pessary with support for treatment. Participants were recruited from a pool of prior research participants who had completed a prospective MRI-based study investigating structural support site failure and the biomechanics of anterior vaginal wall prolapse [10]. In the previous study, all participants had undergone dynamic MRI following a minimum 1-week period of pessary non-use. We selected participants from this prior study to allow for paired comparisons of pelvic floor anatomy and support structures before and after long-term pessary use; these comparisons are being analyzed in a separate, concurrent investigation. Because this was a feasibility study primarily aimed at applying a motion-tracking framework to dynamic MRI analysis, we limited inter-patient variability by including only patients using ring pessaries—the most common pessary type—and those with successful retention to ensure imaging without expulsion during Valsalva.

Each participant underwent a dynamic three-dimensional (3D) MRI for the present study with and without their pessary in situ. Imaging sequences were acquired at rest and during strain, using techniques adapted from previously published protocols [11]. Briefly, midsagittal MR volumes were obtained in the supine position during maximal Valsalva using a Philips Ingenia 3 T scanner with a 15-channel anterior phased array coil. The midsagittal plane was selected because it provides optimal visualization of key pelvic floor landmarks and pessary position. This plane offers a reproducible view while minimizing complexity for this pilot feasibility study, analogous to prior feasibility work using midsagittal transperineal ultrasound to track urethral motion [12]. Participants were coached to perform a Valsalva maneuver that reproduced a prolapse similar in size to that observed during the clinical Pelvic Organ Prolapse Quantification examination (POP-Q), also conducted in the supine position. All Valsalva efforts were performed at maximal effort to ensure that the full extent of the prolapse was visualized on imaging. MR volumes were reviewed to confirm that the degree of prolapse observed during strain corresponded to clinical examination findings. All participants were asked to void prior to both MRI and physical examination to ensure similar comparison.

We used an automated method to track and quantify multiple regions of interest (ROIs) on dynamic MRI. This optical flow–based motion-tracking framework used was previously validated for dynamic tracking of the urethra, demonstrating sub-millimeter accuracy and excellent reproducibility (ICC > 0.9) [12]. The only manual step required is the initial selection of the ROIs. Applying this framework to track pessary rims and other pelvic floor structures ensured that our frame-by-frame measurements reflected true anatomic displacement, enabling robust quantification of device–tissue interaction without manual segmentation of individual frames. A representative example video demonstrating this automated tracking is provided in the supplementary material (Video S1). Detailed descriptions of the image processing and motion tracking algorithms are available in our prior publication [12].

For this pilot analysis, ROIs included the distal (toward hiatus) and proximal (toward vaginal apex) pessary rims, anorectal angle, perineal body, and two reference landmarks—the inferior pubic point and sacrococcygeal joint in the midsagittal plane. The pessary rims were identified as hypointense circles on the midsagittal plane, the perineal body as the region bounded anteriorly by the posterior vaginal wall and posteriorly by the anterior anal wall, and the anorectal angle as the point marking the transition between the anal canal and rectum, defined by the angle change between the two. The bony landmarks were used to perform a rigid transformation of scanner coordinates into the coordinate system of the modified 3D Pelvic Inclination Correction System (3D PICS) [13]. This transformation accounts for variation in participant positioning within the scanner and enables standardized comparisons across participants, allowing objective analysis of pessary motion and dynamic changes in levator and urogenital hiatus dimensions. A single experienced assessor identified all ROIs and landmarks. All transformations were performed using Python programming language (Python Software Foundation, Beaverton, OR).

**Fig. 1:**
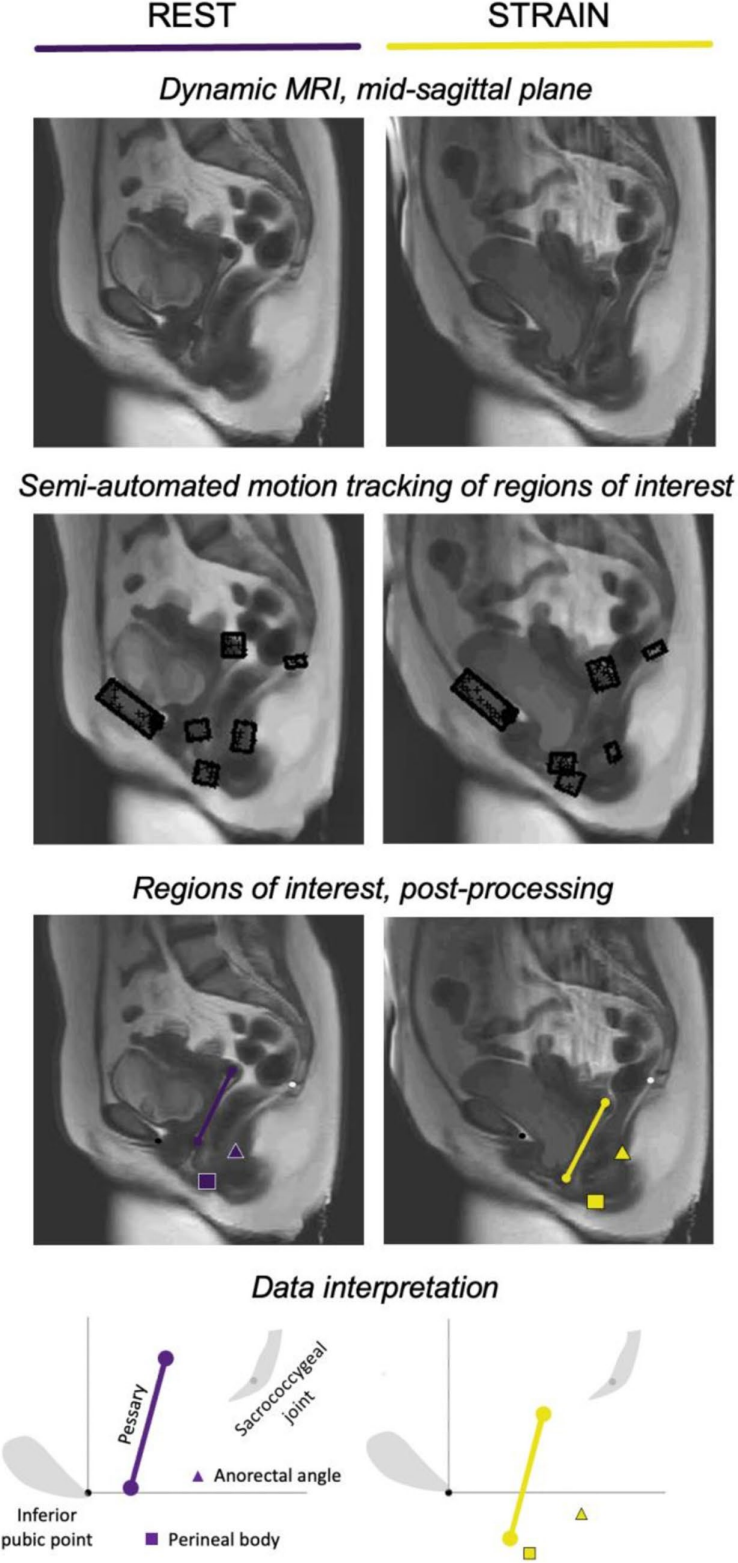
Application of customized motion-tracking software to track regions of interest on dynamic pelvic MRI and convert them into standardized coordinates for data analysis

The motion-tracking software was used to track the translation of each region of interest in the mid-sagittal plane on a frame-by-frame basis while adjusting for changes in pelvic inclination during the Valsalva. The upper left coordinate of each ROI was used as the coordinate point for standardized transformation within the 3D PICS framework (Fig. 1). Urogenital hiatus size was measured as the distance between the pubic bone and the perineal body, while levator hiatus size was defined as the distance between the pubic bone and the anorectal angle. The association between distal pessary translation and hiatus dimensions was assessed using Pearson correlation. A *p* value of < 0.05 was considered statistically significant.

## Results

The median patient age was 66.5 years (range 52–76), and the median duration of pessary use was 3 years (range 2–4). All patients were using a ring pessary, either size 3 or 4 (2.5 to 2.75 in/70 to 76 mm in diameter). All participants reported performing their own pessary self-maintenance. Two of the six patients reported feeling their pessary protrude outside of the vaginal opening less than once per week while in use; the remaining four reported no sensation of pessary protrusion. All patients reported successful pessary retention.

**Fig. 2:**
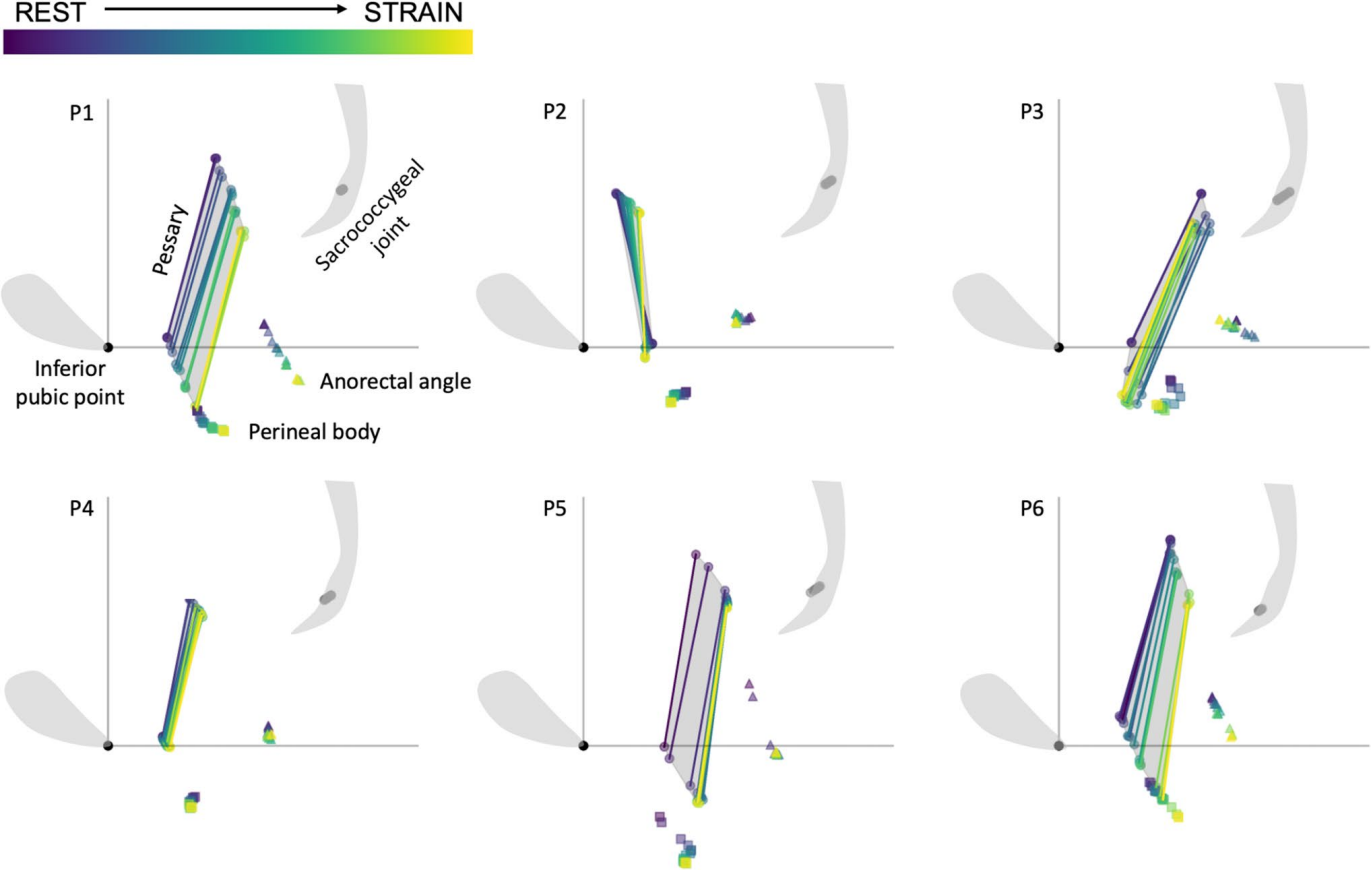
Dynamic motion tracking of the ring pessary, perineal body, and anorectal angle using standardized Pelvic Inclination Correction System (PICS) coordinates for cross-subject comparison of six study participants (P1–P6)

On resting MRI, the distal edge of the pessary was positioned posterior and inferior to the pubic bone, with elevation of both the anterior and posterior vaginal walls observed in all patients. In one case, the proximal edge of the pessary was located in the anterior fornix. Automated tracking of all regions of interest on dynamic MRI was successful for all six patients. Dynamic changes in pessary position and orientation, as well as associated translation of the perineal body and anorectal angle during strain, are illustrated in Fig. 2.

When considering only the start and end frames, the correlation between the distal pessary edge displacement and urogenital hiatus size was strong and approached significance (*r* = 0.81 [95% CI −0.01 to 0.98], *p* = 0.051), but no significant correlation was found for levator hiatus size (*r* = 0.73 [−0.20 to 0.97], *p* = 0.1). However, frame-by-frame motion tracking revealed a stronger significant correlation between the distal pessary edge and urogenital hiatus size (*r* = 0.96 [0.94–0.97], *p* < 0.01) as well as levator hiatus size (*r* = 0.86 [0.81–0.91], *p* < 0.01).

## Discussion

In this prospective pilot study of six patients using ring pessaries with support for the treatment of pelvic organ prolapse, we applied a motion-tracking framework that successfully captured frame-by-frame translation of the pessary and adjacent pelvic structures during strain on dynamic pelvic MRI. Compared to analyses limited to only the initial and final frames of dynamic MRI sequences, this frame-by-frame approach revealed stronger correlations between descent of the distal edge of the pessary and enlargement of both the urogenital and levator hiatuses, supporting our hypothesis. The observation that pessary movement closely parallels downward displacement of the pelvic floor highlights the strong mechanical relationship between these structures.

Despite widespread clinical use, pessary design and practice have changed little over the past several decades. Fitting remains a largely trial-and-error process, constrained by a limited set of historically based designs that were developed without biomechanical insight and are mass-produced without potential for customization. Recent advancements in additive manufacturing (i.e., 3D printing) and a growing interest in novel device mechanics present an opportunity to improve pessary function and usability. Several new pessary designs that enable customization and facilitate improved ease-of-use have recently received US Food and Drug Administration clearance and are becoming available to patients [14, 15]. However, as innovation in pelvic floor devices accelerates, fundamental questions about how pessaries work—and critically, why they fail—remain unanswered. In the absence of a biomechanical foundation, emerging devices are being developed with limited guidance—similar to designing a hip implant without understanding joint articulation or the mechanics of movement it must support.

MRI is emerging as an important tool to address these knowledge gaps. Prior research by Triepels, Boogaard, Notten, and colleagues has used dynamic MRI to examine anatomical factors associated with successful and unsuccessful pessary fitting, identifying sacrococcygeal and pubococcygeal angles, in addition to known clinical factors such as total vaginal length and genital hiatus size, as predictors of fitting success [15]. Their group also analyzed pessary translation and rotation, noting that unsuccessfully fitted pessaries tended to be positioned more caudally at rest and showed greater rotation during Valsalva [16]. MRI studies by van den Noort, Grob, and colleagues has explored pessary position in both supine and upright orientations, with the upright position providing more physiologic insight into pelvic floor mechanics during typical daily function [16]. While these studies have begun to shed light on pessary mechanics, most rely on comparisons between just two frames (e.g., resting and maximal Valsalva frames, or supine and upright frames), limiting the ability to understand real-time dynamic interactions between the pessary and pelvic floor during physiologic loading. This is akin to studying the kinematics of a bowling ball knocking down pins by comparing only two still images: one with all pins standing and one with all pins down, rather than analyzing real-time footage of the ball’s impact.

The motion-tracking framework used in this study helps bridge that gap by enabling detailed analysis of pessary and pelvic floor structure kinematics. In this pilot, we focused on tracking motion in the midsagittal plane, as this is the most commonly studied plane in pessary biomechanics and provided a feasible starting point for software development. With this foundation in place, we can now extend analyses to orthogonal planes and compare findings across dynamic sequences with and without a pessary, enabling hypothesis generation and testing regarding pessary mechanisms of action. By providing quantitative, reproducible metrics of device–tissue interaction, this motion-tracking system could guide personalized pessary fitting strategies, inform design optimization for novel devices, and ultimately improve patient comfort, retention rates, and prolapse symptom relief.

Our finding that distal pessary descent correlates strongly with enlargement of the urogenital and levator hiatuses is intuitive and an apparent observation when viewing dynamic MRI in real time (Video 1). However, the significance lies in the ability to now quantitatively analyze less visually obvious interactions, and to study how these dynamics vary with different pessary shapes and sizes, and across patients with different pelvic floor anatomies.

While the primary objective of this study was to validate the motion-tracking framework in dynamic MRI, our findings also contribute to existing literature on pessary mechanics. The consistent posterior–inferior positioning of the distal pessary edge supports prior evidence that the pubic bone plays a limited role in retention, emphasizing the importance of the genital hiatus as the primary support structure. Future studies will be needed to clarify causal relationships between pessary descent, hiatus size, and other structural variations such as levator ani shape or avulsion.

Lastly, similar to previous studies assessing pessary position at rest, we observed heterogeneity in pessary positioning within the vaginal canal. Each of the six individuals’ pessary had a unique relationship to perineal body and anorectal angle and pattern of movement. Of interest, one patient had the proximal pessary rim seated in the anterior fornix. This finding raises questions about the hypothesized “uterine lever mechanism,” which posits that downward loading on the proximal pessary edge elevates the distal edge to support prolapse [7, 16, 17]. Our findings suggest that full-length positioning along the anterior vaginal wall may also provide effective symptom relief, which may be particularly relevant for patients with a significantly elongated anterior vaginal wall.

This study has several notable strengths, including the use of a standardized coordinate system for quantitative analysis and the consistent application of imaging techniques, measurement protocols, and analytic strategies across all participants by an experienced, interdisciplinary research team. The techniques developed here may also have applications in understanding the mechanics of pelvic floor devices beyond vaginal pessaries for prolapse, such as a novel vaginal orthosis being designed to facilitate postoperative wound healing following native tissue reconstructive pelvic floor surgery [18]. The near real-time and automated nature of this framework also enables potential clinical applications, where manual tracking of each frame would not be feasible.

Nevertheless, these findings should be interpreted in light of several limitations. Although the software enables automated frame-by-frame tracking, regions of interest must still be manually selected by a trained assessor. However, for a trained assessor, this is a straightforward process that takes less than 1 min per patient. With future development and integration of artificial intelligence, full automation and segmentation may be possible [19]. The small sample size of six patients—all successful ring pessary users—was sufficient to demonstrate feasibility but limits generalizability; larger and more diverse populations are needed to better understand pessary function and failure. Future studies that include both successful and unsuccessful pessary users will allow for evaluation of how anatomic variation and user factors influence pessary migration, and may also leverage dynamic upright MRI to more closely replicate physiologic loading under gravity. Finally, analysis was limited to the midsagittal plane, where key structures are easily visualized. Application to other planes may require adapting the software to identify less distinct or shape-based structures; this is currently under investigation.

In conclusion, this pilot study demonstrates the feasibility of an automated motion-tracking framework for evaluating pessary and pelvic floor kinematics on dynamic MRI. By enabling frame-by-frame analysis, this method reveals temporal relationships between pessary motion and hiatus dynamics that are not captured by start–end frame comparisons. This approach offers a novel, quantitative tool for advancing biomechanical understanding of pessary function and informing future pelvic floor device design.

## Supplementary Information

Below is the link to the electronic supplementary material.

Supplementary file1 Video 1. Left: Dynamic MRI in the midsagittal plane with a ring pessary in situ. Right: Selected regions of interest (ROIs), including the pubic symphysis, sacrococcygeal joint, anorectal angle, perineal body, and anterior/posterior pessary rims. Motion tracking captures frame-by-frame displacement of these ROIs in real time. (MOV 640 KB)

## Declarations

### Prior Presentation

This study was presented at the 2025 International Urogynecological Association/European Urogynecological Association Joint Annual Meeting in Barcelona, Spain.

## References

[1] Wu JM, Vaughan CP, Goode PS, et al. Prevalence and trends of symptomatic pelvic floor disorders in U.S. women. Obstet Gynecol. 2014;123:141–8. 24463674 10.1097/AOG.0000000000000057 PMC3970401

[2] Panman CMCR, Wiegersma M, Kollen BJ, et al. Effectiveness and cost-effectiveness of pessary treatment compared with pelvic floor muscle training in older women with pelvic organ prolapse: 2-year follow-up of a randomized controlled trial in primary care. Menopause. 2016;23:1307–18. 27504918 10.1097/GME.0000000000000706

[3] Ben ÂJ, van der Vaart LR, E Bosmans J, et al. Cost-effectiveness of pessary therapy versus surgery for symptomatic pelvic organ prolapse: an economic evaluation alongside a randomised non-inferiority controlled trial. BMJ Open. 2024;14:e075016. 10.1136/bmjopen-2023-075016 PMC11086579 38692718

[4] Clemons JL, Aguilar VC, Tillinghast TA, et al. Risk factors associated with an unsuccessful pessary fitting trial in women with pelvic organ prolapse. Am J Obstet Gynecol. 2004;190:345–50. 14981372 10.1016/j.ajog.2003.08.034

[5] Yurteri-Kaplan LA, Meyn L, Moalli PA, et al. Outcomes of pessary use at 1 year in women treated for pelvic organ prolapse in a large multicenter registry: developed by the Pelvic Floor Disorders Registry. Urogynecology. 2022;28:800–10. 36409637 10.1097/SPV.0000000000001279

[6] Jones KA, Harmanli O. Pessary use in pelvic organ prolapse and urinary incontinence. Rev Obstet Gynecol. 2010;3:3–9. 20508777 PMC2876320

[7] Hong CX, Meer E, Cioban M, et al. Position and orientation of vaginal pessaries in situ on magnetic resonance imaging. Int Urogynecol J. 2022;33:369–76. 34132867 10.1007/s00192-021-04888-7

[8] Boogaard LL, Triepels CPR, Verhamme LM, et al. Location and motion of vaginal pessaries in situ in women with successful and unsuccessful pessary treatment for pelvic organ prolapse. Int Urogynecol J. 2023;34:2293–300. 37119269 10.1007/s00192-023-05555-9 PMC10506932

[9] Triepels CPR, Boogaard LL, Fütterer JJ, et al. Explorative identification of anatomical parameters associated with successful pessary fitting in pelvic organ prolapse using dynamic magnetic resonance imaging. J Clin Med. 2024;13:4819. 39200962 10.3390/jcm13164819 PMC11355653

[10] Hong CX, Nandikanti L, Shrosbree B, et al. Variations in structural support site failure patterns by prolapse size on stress 3D MRI. Int Urogynecol J. 2023;34:1923–31. 36802015 10.1007/s00192-023-05482-9 PMC10577811

[11] Larson KA, Luo J, Guire KE, et al. 3D analysis of cystoceles using magnetic resonance imaging assessing midline, paravaginal, and apical defects. Int Urogynecol J. 2012;23:285–93. 22068322 10.1007/s00192-011-1586-x PMC3593601

[12] Kalayeh K, Fowlkes JB, Sack BS, et al. A new automated ultrasound quantification of urethral mobility for stress urinary incontinence: a feasibility study. J Ultrasound Med. 2025;44:1213–27. 40095237 10.1002/jum.16676 PMC12149724

[13] Matter L, Hebeisen M, Beintner-Skawran S, et al. MRI characterization of pelvic floor ligaments in nulliparous women: technique development and morphometry within the 3D pelvic inclination correction system (3D-PICS). Eur J Radiol. 2024;173:111351. 38340570 10.1016/j.ejrad.2024.111351

[14] Hong CX, Zhang S, Eltahawi A, et al. Patient-specific pessaries for pelvic organ prolapse using three-dimensional printing: a pilot study. Urogynecol. 2023;29:732–9. 10.1097/SPV.0000000000001346 PMC10476593 36946908

[15] Strohbehn K, Wadensweiler PM, Richter HE, et al. Effectiveness and safety of a novel, collapsible pessary for management of pelvic organ prolapse. Am J Obstet Gynecol. 2024;231:271.e1-271.e10. 38761837 10.1016/j.ajog.2024.05.009 PMC11283992

[16] van der Steen A, Simonis FFJ, Grob ATM. Pelvic organ prolapse quantification after pessary removal: the use of upright MRI in POP research. Int Urogynecol J. 2025. 10.1007/s00192-025-06182-2. 40464913 10.1007/s00192-025-06182-2 PMC12356738

[17] Atnip SD. Pessary use and management for pelvic organ prolapse. Obstet Gynecol Clin North Am. 2009;36:541–63. 19932415 10.1016/j.ogc.2009.08.010

[18] Cadena MC, Hong CX, Blokker A, et al. Vaginal orthosis after native tissue reconstructive surgery: design and phase 0. Urogynecology. 2025;31:309–14. 39715052 10.1097/SPV.0000000000001628

[19] Mylonas A, Booth J, Nguyen DT. A review of artificial intelligence applications for motion tracking in radiotherapy. J Med Imaging Radiat Oncol. 2021;65(5):596–611. 34288501 10.1111/1754-9485.13285

